# Association of cerebral microvascular perfusion and diffusion dynamics detected by intravoxel incoherent motion-diffusion weighted imaging with initial neurological function and clinical outcome in acute ischemic stroke

**DOI:** 10.7717/peerj.12196

**Published:** 2021-09-16

**Authors:** Fei Chen, Zhenyu Dai, Lizheng Yao, Congsong Dong, Haicun Shi, Weiqiang Dou, Wei Xing

**Affiliations:** 1 Department of Radiology, The Third Affiliated Hospital of Soochow University, Changzhou, Jiangsu, China; 2 Department of Radiology, Yancheng Third People’s Hospital, Yancheng, Jiangsu, China; 3 Department of Neurology, Yancheng Third People’s Hospital, Yancheng, Jiangsu, China; 4 GE Healthcare, MR Research China, Bejing, China

**Keywords:** Stroke, Intravoxel incoherent motion, Diffusion weighted imaging, Perfusion, Magnetic resonance imaging

## Abstract

**Background:**

This work aimed to explore the association of cerebral microvascular perfusion and diffusion dynamics measured by intravoxel incoherent motion (IVIM) imaging with initial neurological function and clinical outcome in acute stroke.

**Methods:**

In total, 39 patients were assessed with admission National Institutes of Health Stroke Scale (NIHSS) and day-90 modified Rankin Scale (mRS). The parametrical maps of IVIM were obtained, including apparent diffusion coefficient (ADC), pseudo-diffusion coefficient (D*), true diffusion coefficient (D) and perfusion fraction (f). The fD* was the product of f and D*. Moreover, the ratios of lesioned/contralateral parameters (rADC, rD, rD*, rf and rfD*) were also obtained. The differences of these parameters between the poor outcome group and good outcome group were evaluated. Partial correlation analysis was used to evaluate the correlations between the admission NIHSS/day-90 mRS and each parameter ratio, with lesion volumes controlled.

**Results:**

The ADC, D, D*, f and fD* values of lesions were significantly reduced than those of the contralateral regions. The rADC and rD were significantly decreased in the poor outcome group than good outcome group (all *p* < 0.01). With lesion volume controlled, rADC showed a weak negative correlation (*r* = −0.340, *p* = 0.037) and a notable negative correlation (*r* = −0.688, *p* < 0.001) with admission NIHSS score and day-90 mRS score, respectively. In addition, rD showed a strong negative correlation (*r* = −0.731, *p* < 0.001) with day-90 mRS score.

**Conclusion:**

Significant negative correlations were revealed between IVIM derived diffusion dynamics parameters and initial neurological function as well as clinical outcome for patients with acute ischemic stroke. IVIM can be therefore suggested as an effective non-invasive method for evaluating the acute ischemic stroke.

## Introduction

Stroke, as a major health problem, has become the leading cause of death in China as well as the second leading cause of death in the world (Zhou et al., 2019). In China, acute ischemic stroke accounts for 70% of stroke, and one-year mortality rate is as high as 33% (Wang et al., 2017a, 2017b). It is a critical disease in the department of neurology and seriously endangers patients health and quality of life.

Neuroimaging is an important tool in clinical diagnosis and treatment evaluation of acute ischemic stroke. Computed tomography, magnetic resonance diffusion weighted imaging (DWI) and perfusion weighted imaging (PWI) have been commonly used to diagnose acute stroke and to evaluate its therapeutic effect and clinical prognosis (Scheldeman et al., 2020; Suh et al., 2019; Zhu et al., 2019). Among the neuroimaging techniques mentioned above, previous studies showed that DWI is the most valuable approach for the detection of acute stroke (Heiss & Zaro-Weber, 2019; Sagnier & Sibon, 2019; Scheldeman et al., 2020; Simonsen et al., 2015; Wolman et al., 2018; Wouters et al., 2016). The ischemic lesions exhibit themselves *via* evident hyperintensity on DWI, and accompanied by a decrease in apparent diffusion coefficient (ADC) values (Liu et al., 2019; Wolman et al., 2018). Restricted diffusion of ischemic lesion has been attributed to the restricted motion of molecular diffusion of water and microvascular perfusion in the capillary network, separately (Thiel et al., 2019; Zhu et al., 2019). In stroke studies, differentiating the mixed diffusion components is essential for accurate evaluation of the diffusion and perfusion characteristics of ischemic lesions. Conventional DWI with derived ADC however, cannot achieve this goal.

Intravoxel incoherent motion (IVIM)-DWI, as an attractive multi-b values diffusion imaging technique, is able to separate tissue diffusivity and perfusion-related diffusivity (Liu et al., 2019; Thiel et al., 2019; Togao et al., 2018; Yao et al., 2016; Zhu et al., 2019). With bi-exponential model, the resultant IVIM parameters have been reported to closely relate with ADC derived by conventional DWI or perfusion metrics obtained in perfusion weighted imaging. More specifically, the true diffusion coefficient (D) is positively related to ADC, and the pseudo-diffusion coefficient (D*) is negatively correlated with the time to peak or mean transit time. Moreover, the perfusion fraction (f) shows close positive relationship with cerebral blood volume (CBV), this relationship also exists between the product of f and D* (fD*) and cerebral blood flow (CBF). The perfusion-related parameters (f, D* and fD*) allow for monitoring the density and integrity of the capillary network (Federau et al., 2019; Thiel et al., 2019; Togao et al., 2018; Zhu et al., 2019). IVIM has been employed in a number of studies for tumor grading (Togao et al., 2018), recurrence prediction (Detsky et al., 2017) and stroke assessment (Zhu et al., 2019) and so on. The findings reported in these stroke studies were however not fully agreed in D* change at ischemic lesion (Suo et al., 2016; Yao et al., 2016), and the detection of ischemic penumbra using parameter f (Federau et al., 2019; Zhu et al., 2019).

So far, a number of magnetic resonance imaging (MRI) studies have attempted to explore the associations between DWI or PWI of ischemic lesions and stroke outcome to further predict the prognosis of stroke at early stage of onset (de Havenon et al., 2017; Liu et al., 2019; Nadareishvili et al., 2019; Thamm et al., 2019; Yu et al., 2018). However, the relationship between both microvascular perfusion and diffusion dynamics measured by IVIM and initial neurological function as well as clinical outcome remains uncertain for acute ischemic stroke patients.

Hence, the main purpose of this work was to explore the applicability of IVIM-DWI in the detection of both microvascular perfusion and diffusion dynamics for acute ischemic stroke patients and to evaluate the relationships of the resultant diffusion-related parameters (ADC and D) and microvascular perfusion-related parameters (f, D* and fD*) with initial neurological function and clinical outcome for patients with acute ischemic stroke. To achieve this goal, multi-b value IVIM data were acquired and analyzed with a biexponential model.

## Methods

### Patients

**Figure 1:**
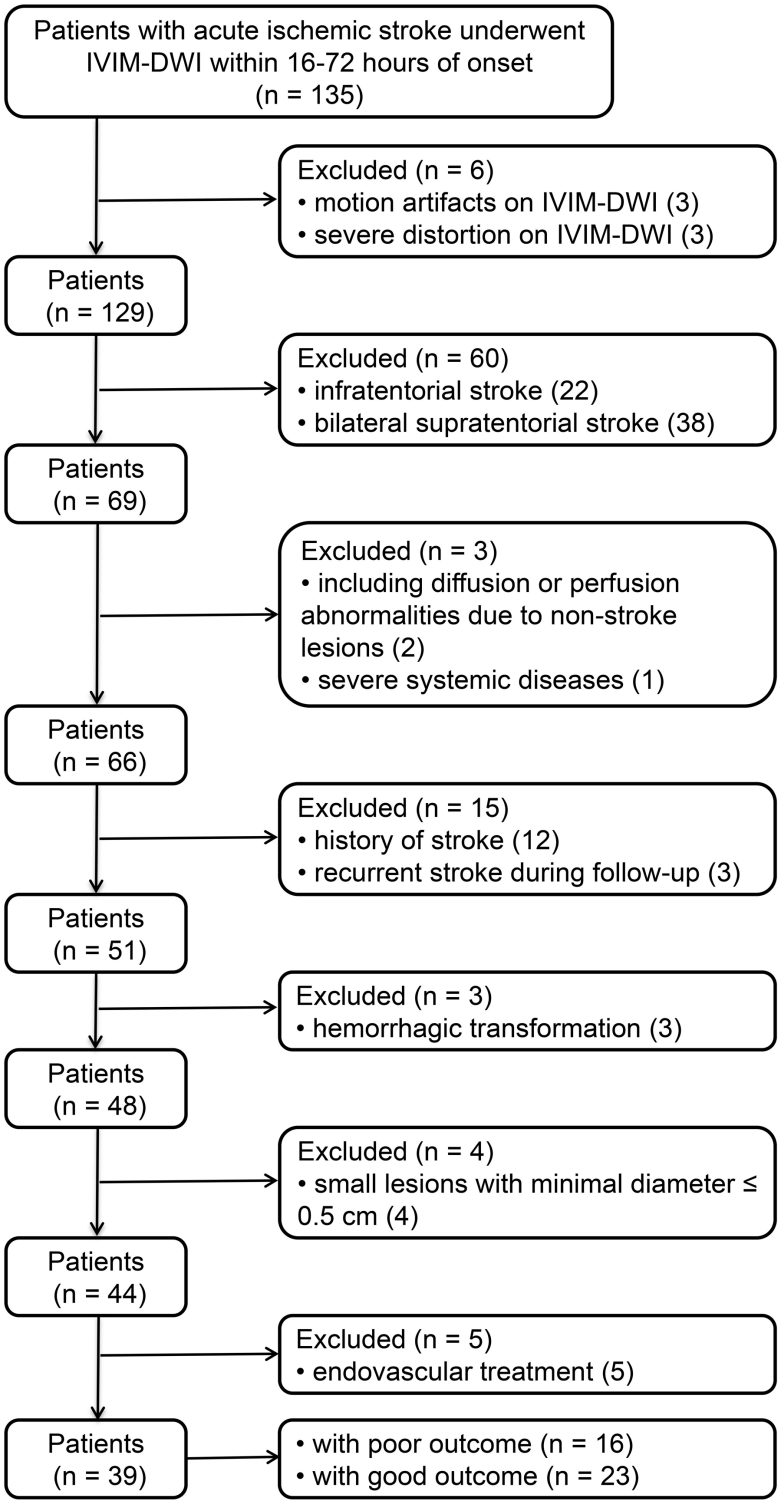
Patient distribution. IVIM-DWI: intravoxel incoherent motion diffusion weighted imaging.

This study was approved by the Ethics Committee of Yancheng Third People’s Hospital (No. 2015092816), and was adherent to the Declaration of Helsinki. All patients or next of kin signed the informed consent. A total of 135 patients with acute ischemic stroke in our hospital from July 2014 to January 2019, underwent conventional MRI and IVIM measurement within 6–48 h post-onset. Exclusion criteria (Fig. 1) were defined and applied for these patients: (1) infratentorial stroke or bilateral supratentorial stroke, (2) history of stroke or recurrent stroke during follow-up, (3) minimal diameter of lesion ≤0.5 cm, (4) hemorrhagic transformation, (5) systemic intravenous thrombolytic therapy or endovascular treatment, (6) severe artifacts on IVIM, (7) diffusion or perfusion abnormalities due to non-stroke lesions and (8) severe systemic diseases.

In this study, 39 patients were thus finally included (Fig. 1). Standard clinical treatment were applied for all patients to achieve an optimal functional improvement. Patient demographic and clinical characteristics were recorded from the electronic medical record and shown in Table 1. Variables noted were: age, sex, dominant hemisphere, time from stroke onset to MRI, hypertension, alcohol abuse, smoking, atrial fibrillation, diabetes mellitus, hyperlipidemia.

**Table 1:** Demographic and clinical characteristics of the patient groups of poor and good outcome.

	Poor outcome (*n* = 16, mRS ≤ 2)	Good outcome (*n* = 23, 3 ≤ mRS ≤ 6)	*p* value
Age (years)^a^	71 ± 14	68 ± 12	0.441
Sex, male/female	5/11	14/9	0.105
Dominant hemisphere, *n* (%)	10 (63)	10 (43)	0.333
Time from stroke onset to MRI (hour)^a^	28 ± 14	31 ± 11	0.412
Admission NIHSS^a^	13 ± 6	5 ± 3	<0.001
Smoking, *n* (%)	7 (44)	6 (26)	0.312
Alcohol abuse, *n* (%)	6 (38)	6 (26)	0.498
Hypertension, *n* (%)	12 (75)	11 (48)	0.111
Atrial fibrillation, *n* (%)	5 (31)	3 (13)	0.235
Diabetes mellitus, *n* (%)	6 (38)	4 (17)	0.264
Hyperlipidemia, *n* (%)	7 (44)	5 (22)	0.174
DWI lesion volume (ml)^b^	37.7 (16.3–239.4)	3.2 (1.5–7.5)	<0.001

**Notes:**

a Mean ± standard deviation.

b Median (interquartile range).

MRI: Magnetic resonance imaging, DWI: Diffusion weighted imaging, NIHSS: National Institutes of Health Stroke Scale, mRS: modified Rankin Scale.

Admission National Institutes of Health Stroke Scale (NIHSS), representing the initial neurological function, was used to rate each patient at baseline before any intervention. Clinical outcome was assessed using the day-90 modified Rankin Scale (mRS). All recruited patients were thus divided into the poor outcome group (3 ≤ day-90 mRS ≤ 6) and good outcome group (day-90 mRS ≤ 2).

### Magnetic resonance imaging protocol

All patients underwent MRI examinations on a 3.0 T unit (HDxt; GE Healthcare, Waukesha, WI, USA) equipped with an eight channel head coil. MRI protocol of stroke included 3D time-of-flight angiography, T_1_ weighted imaging, T_2_ weighted-fluid attenuated inversion recovery-imaging and IVIM sequences. According to the scan parameters applied previously (Federau et al., 2012, 2014), the imaging parameters of IVIM were set as follows: TR = 5,825 ms, TE = 94 ms, FOV = 24 × 24 cm^2^, slice thickness/gap = 5 mm/1.5 mm, matrix size = 128 × 128, acceleration factor = 2, *b* values = 0, 10, 20, 40, 80, 110, 140, 170, 200, 300, 400, 500, 600, 800, 1,000 s/mm^2^, and acquisition time = 7 min 11 s.

### Image processing

Post-processing of all data were implemented through a vendor-provided workstation (AW 4.5; GE Healthcare, Waukesha, WI, USA). Firstly, a motion correction was done for IVIM images before the calculation of parametric maps. Then, a two-step fitting procedure was used to fit IVIM data. At the first step, D was derived from b-values > 200 s/mm^2^ based on a mono-exponential model of least-squares linear fitting (S_b_/S_0_ = exp^-bD^), where S_0_ and S_b_ are the signal intensity at b-value of 0 s/mm^2^ and at a specific b value, respectively (Yao et al., 2016). In the second step, f and D* values were obtained from all b-values based on the bi-exponential IVIM model of Levenberg-Marquardt non-linear fitting [S_b_/S_0_ = f × exp^-bD*^ + (1 − f) × exp^−bD^], while keeping D fixed (Suo et al., 2016). The fD* was further calculated as the product of D* and f. In order to avoid the influence of noise and cerebrospinal fluid, the bound constraints for physiological D* and f were set as 0 ≤ D* ≤ 0.05 mm^2^/s and 0 ≤ f ≤ 0.3, respectively (Federau et al., 2012). The bound constraints for D* and f were set simultaneously, one of them was outside of the range, all other parameters in the specific voxel would be excluded instead of set them to zero. Moreover, the ADC value was also fitted by a mono-exponential model (S_b_/S_0_ = exp^-bADC^) using all b-values, where S_0_ and S_b_ are the signal intensity at b-value of 0 s/mm^2^ and at a specific b value (Hu et al., 2014).

All lesion volumes were automatically displayed by delineating the hyperintensities on each slice of diffusion images (b-value = 1,000 s/mm^2^). Regions of interest (ROIs) were manually drew by two neuroradiologists, who were blinded to clinical data, in the ischemic core. The mirror ROIs were then placed in the contralateral hemisphere while these ROIs should avoid notable cerebral spinal fluid or large vessels. The ROIs were consistent across all metric maps. The averaged values of each parameter and lesion volumes measured by both neuroradiologists were used for further statistical analysis. In addition, the ratios of lesioned/contralateral parameters (rADC, rD, rD*, rf and rfD*) were calculated for inter-group comparison and correlation analysis.

### Statistical analysis

All statistical analyses were performed using SPSS software (Version 20.0; IBM, Armonk, NY, USA). The intraclass correlation coefficient (ICC) was used to evaluate the inter-observer agreement of IVIM parameters and lesion volumes, and was considered to be poor (0 ≤ ICC ≤ 0.4), weak (0.4 < ICC ≤ 0.75), fair to good (0.75 < ICC ≤ 0.9), or excellent (0.9 < ICC ≤ 1). Normality test was analyzed using the Kolmogorov–Smirnov test. A two-tailed paired t-test was used to compare the IVIM parameters between stroke and contralateral hemisphere. An independent sample t-test was used to compare the variables with normal distribution (mean ± standard deviation (SD)), the Mann-Whitney U-test was used to compare the lesion volume (median (interquartile range)), and the Fisher’s exact test was used to compare a series of clinical characteristics between good outcome group and poor outcome group. In addition, partial correlation analysis was employed between the IVIM parameters and initial neurological function as well as the clinical outcome, with the lesion volume controlled as a confounder. Statistical significance was defined at *p* < 0.05.

## Results

### General clinical characteristics

A total of 135 patients [65 males, age = 69 ± 12 (mean ± SD) years] were collected from July 2014 to January 2016, excluding 96 patients based on the exclusion criteria (Fig. 1). Finally, 39 patients (19 males, age = 69 ± 13 (mean ± SD) years) were included in this study. Evaluating with the day-90 mRS, these patients were further divided into group of poor outcome (*n* = 16) and good outcome (*n* = 23). Table 1 summarizes the demographic and clinical characteristics of patients.

### General features of parametric maps

**Figure 2:**
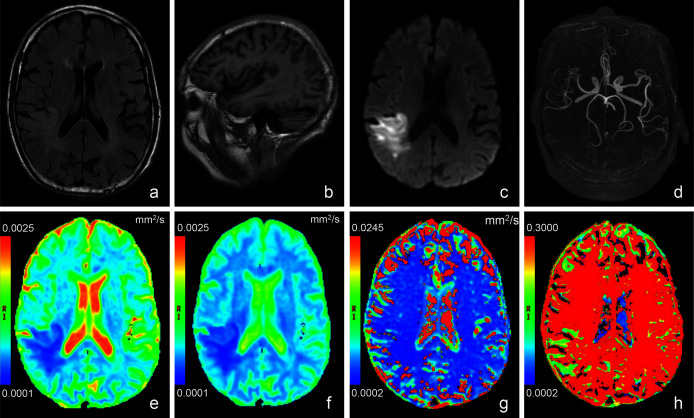
Images of a representative patient (male, 78 years old, lesion volume: 41.5 ml, time from stroke onset to MRI: 24 h, National Institutes of Health Stroke Scale on admission: 13, day-90 modified Rankin Scale: 3) with acute stroke in the *r*. T_2_ weighted fluid attenuated inversion recovery imaging (A) showed a slightly hyperintense signal in the lesion. No obvious abnormal signal was found in T_1_ weighted imaging (B). (C) was a diffusion image (*b* value = 1,000 s/mm^2^) of intravoxel incoherent motion-diffusion weighted imaging, and it demonstrated the infarct lesion with apparent abnormal high signal. 3D time-of-flight magnetic resonance angiography (D) showed that the distal branches of the middle cerebral artery in the lesion area were more sparse than those on the contralateral area. Compared with the normal brain tissue, the infarct lesion in this case showed significantly lower signal on apparent diffusion coefficient (E), true diffusion coefficient (F) and perfusion fraction (H) maps, as well as slightly lower signal on pseudo-diffusion coefficient (G) map.

**Figure 3:**
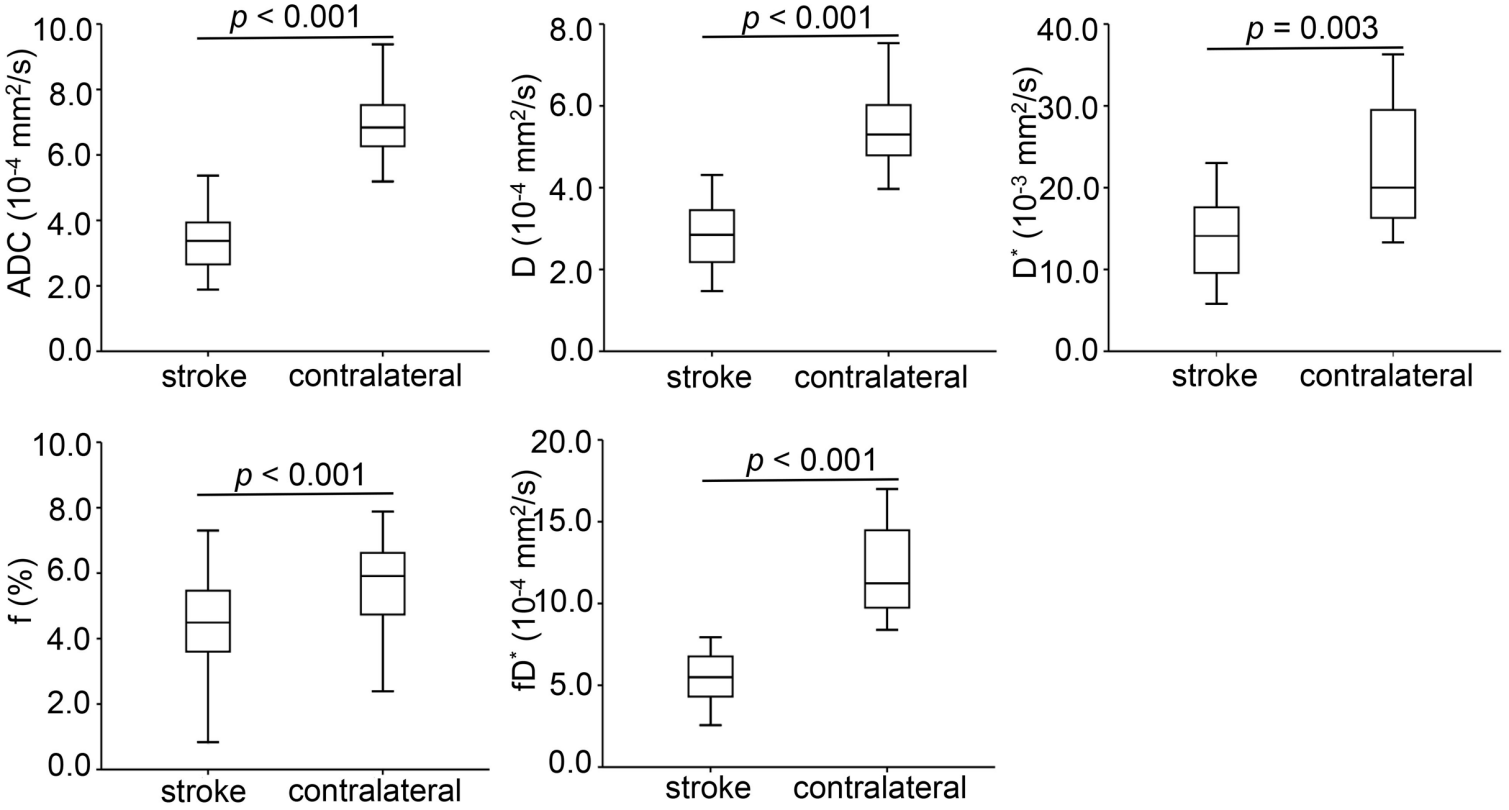
Box-and-whisker plots of apparent diffusion coefficient and intravoxel incoherent motion diffusion weighted imaging derived parameters in the stroke and healthy contralateral areas.

Conventional anatomic MRI and multiple parametric maps of IVIM were shown for a representative patient with acute ischemic stroke (Fig. 2). Inter-observer agreement for the quantitative analysis of all IVIM derived parameters were evaluated as fair to good, and for the lesion volumes was excellent. More precisely, the ICC values of D*, f, D,ADC and lesion volumes were 0.770, 0.773, 0.880, 0.891 and 0.995, respectively. Table 2 and Fig. 3 showed the results of the quantitative analysis (*n* = 39) for each parametric maps. The ADC (3.42 ± 0.93 *vs*. 7.28 ± 1.64 × 10^−4^ mm^2^/s, *p* < 0.001), D (2.84 ± 0.81 *vs*. 5.57 ± 1.00 × 10^-4^ mm^2^/s, *p* < 0.001), D* (14.65 ± 8.45 *vs*. 22.09 ± 7.94 × 10^−3^ mm^2^/s, *p* = 0.003), f (4.42 ± 1.69 *vs*. 5.70 ± 1.24 %, *p* < 0.001) and fD* (6.20 ± 3.23 *vs*. 12.79 ± 5.09 × 10^−4^ mm^2^/s, *p* < 0.001) values of lesions were significantly reduced than those of the contralateral regions.

**Table 2:** Comparison of IVIM derived parameters between stroke and contralateral hemisphere.

Parameter	Stroke	Contralateral	*p* value
ADC (10^−4^ mm^2^/s)	3.42 ± 0.93	7.28 ± 1.64	<0.001
D (10^−4^ mm^2^/s)	2.84 ± 0.81	5.57 ± 1.00	<0.001
D* (10^−3^ mm^2^/s)	14.65 ± 8.45	22.09 ± 7.94	0.003
f (%)	4.42 ± 1.69	5.70 ± 1.24	<0.001
fD* (10^−4^ mm^2^/s)	6.20 ± 3.23	12.79 ± 5.09	<0.001

**Note:**

IVIM: intravoxel incoherent motion, ADC: apparent diffusion coefficient, D: true diffusion coefficient, D*: pseudo-diffusion coefficient, f: perfusion fraction, fD*: the product of f and D*. Data are presented as mean ± standard deviation (across 39 cases). Statistical significance, *p* < 0.05.

### Inter-group comparison of parameters between poor outcome and good outcome

**Figure 4:**
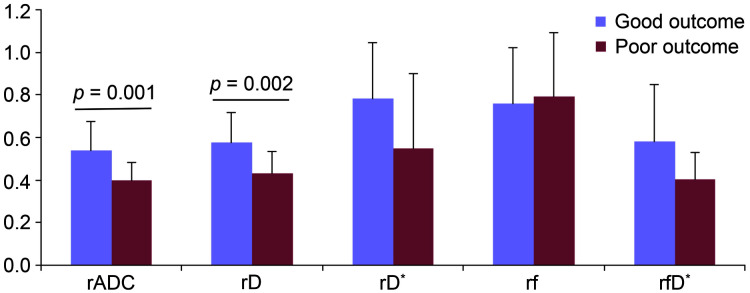
Bar graphs of the parameters between the group of good outcome and poor outcome. The rADC, rD, rD*, rf and rfD* represent the lesioned/contralateral ratios of apparent diffusion coefficient, true diffusion coefficient, pseudo-diffusion coefficient, perfusion fraction, and the product of f and D*, respectively.

The lesion volume [37.7 (16.3–239.4) *vs*. 3.2 (1.5–7.5) ml, *p* < 0.001] was significantly larger in the patient of poor outcome group if compared to patient of good outcome group. As a consequence, the admission NIHSS score (5 ± 3 *vs*. 13 ± 6, *p* < 0.001) was significantly lower accordingly. Other clinical characteristics were not statistically different between both groups (Table 1). In order to eliminate the inter-subject effect on the results, the lesioned/contralateral parameter ratios (rADC, rD, rD*, rf and rfD*) were used for comparing both groups. The rADC (0.40 ± 0.08 *vs*. 0.54 ± 0.14, *p* = 0.001) and rD (0.43 ± 0.10 *vs*. 0.58 ± 0.14, *p* = 0.002), as two ratio parameters related to diffusion dynamics, were significantly lower in the patient of poor outcome than of good outcome, nevertheless the ratios of microvascular perfusion parameters (rD*, rf and rfD*) showed comparable values between two groups (Table 3 and Fig. 4).

**Table 3:** Inter-group comparison of IVIM derived parameter ratios between good outcome group and poor outcome group.

Parameter ratio	Good outcome (*n* = 23)	Poor outcome (*n* = 16)	*p* value
rADC	0.54 ± 0.14	0.40 ± 0.08	0.001
rD	0.58 ± 0.14	0.43 ± 0.10	0.002
rD*	0.78 ± 0.27	0.55 ± 0.35	0.142
rf	0.76 ± 0.26	0.79 ± 0.30	0.702
rfD*	0.58 ± 0.27	0.40 ± 0.13	0.116

**Note:**

IVIM: intravoxel incoherent motion, rADC, rD, rD*, rf and rfD*: The lesioned/contralateral ratios of apparent diffusion coefficient, true diffusion coefficient, pseudo-diffusion coefficient, perfusion fraction, and the product of f and D*. Data are presented as mean ± standard deviation. Statistical significance, *p* < 0.05.

### Correlation between IVIM-DWI parameters and initial neurological function as well as clinical outcome

**Figure 5:**
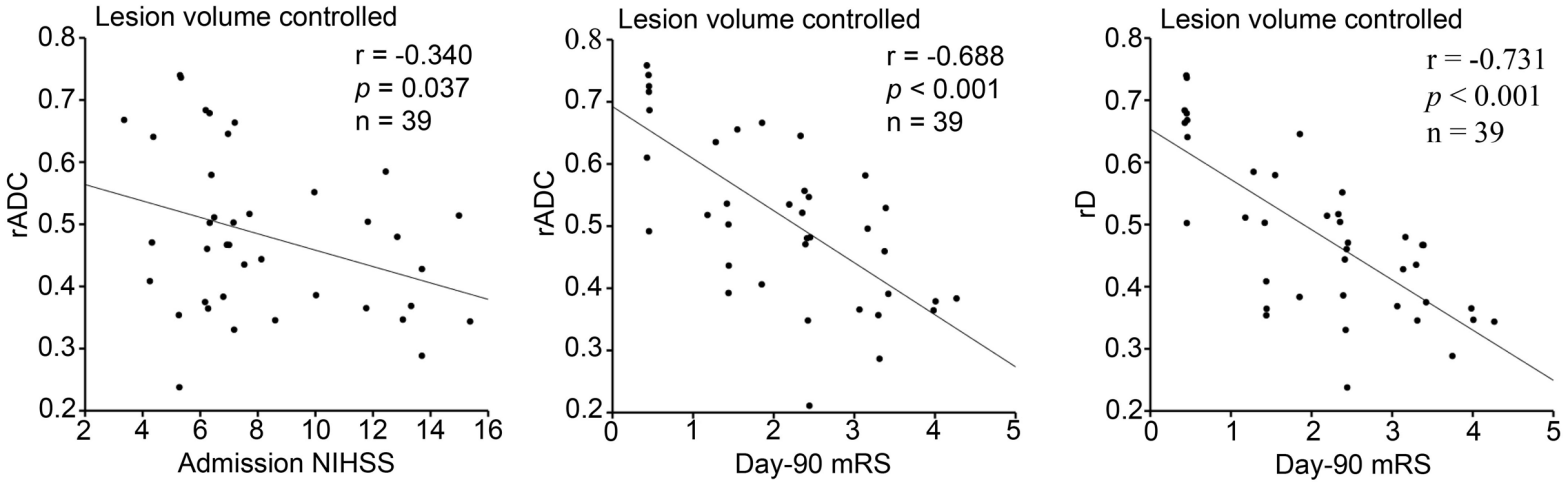
Partial correlation between diffusion parameter ratios and admission NHISS/day-90 mRS scores with lesion volume controlled (*n* = 39). NIHSS: National Institutes of Health Stroke Scale, mRS: modified Rankin Scale. rADC, rD: The lesioned/contralateral ratios of apparent diffusion coefficient, true diffusion coefficient. Statistical significance, *p* < 0.05.

In this study, the initial neurological function representing the primal stroke severity was evaluated by admission NIHSS, and the clinical outcome delegating the final degree of stroke recovery was measured by day-90 mRS. While a confounding factor of lesion volume was controlled, rADC showed a weak negative correlation (r = −0.340, *p* = 0.037) with admission NIHSS score and a notable negative correlation (*r* = −0.688, *p* < 0.001) with day-90 mRS score (Table 4 and Fig. 5). In addition, rD revealed a strong negative correlation (r = −0.731, *p* < 0.001) with day-90 mRS score when lesion volume was controlled (Table 4 and Fig. 5). However, no statistical correlation was observed between cerebral microvascular perfusion related parameter ratios (rD*, rf and rfD*) and initial neurological function, as well as clinical outcome (Table 4).

**Table 4:** Partial correlation between IVIM derived parameter ratios and admission NIHSS scores/Day-90 mRS scores with lesion volume controlled (*n* = 39).

		rADC	rD	rD*	rf	rfD*
Admission NIHSS	*r*	−0.340	−0.262	0.117	−0.085	0.009
	*p* value	0.037	0.117	0.666	0.613	0.973
Day-90 mRS	*r*	−0.688	−0.731	−0.149	−0.057	−0.285
	*p* value	< 0.001	< 0.001	0.583	0.735	0.285

**Note:**

IVIM: intravoxel incoherent motion, NIHSS: National Institutes of Health Stroke Scale, mRS: modified Rankin Scale. rADC, rD, rD*, rf and rfD*: The lesioned/contralateral ratios of apparent diffusion coefficient, true diffusion coefficient, pseudo-diffusion coefficient, perfusion fraction, and the product of f and D*. Statistical significance, *p* < 0.05.

## Discussion

This study was a first attempt to simultaneously explore the associations of cerebral microvascular perfusion and diffusion dynamics with neurological function on admission and clinical outcome of acute ischemic stroke. Using perfusion and diffusion related IVIM parameters, the dynamic changes in ischemic lesions and the difference between good and poor outcome groups were investigated. In addition, we also analyzed the correlation between the corresponding ratio parameters and admission NIHSS score, as well as day-90 mRS score.

In this study, both diffusion-related parameters and microvascular perfusion-related parameters showed significantly decreased values in ischemic lesions than those at contralateral regions (all *p* < 0.01). These findings in acute ischemic stroke assessment were similar to those reported previously (Suo et al., 2016; Yao et al., 2016; Zhu et al., 2019). The decreased ADC and D values indicated restricted diffusion dynamics of the infarct lesion, and can be explained by a decreased Brownian motion caused by cytotoxic oedema occurs in the ischemic lesion. In addition, for perfusion-related parameters, the decreased f, D* and fD* represented lower microvascular perfusion in the infarct lesion. As a consequence of microvascular abnormalities, the capillary transport of oxygen and metabolites were declining, these parameters were correlated with the absence of cerebral blood volume and blood flow in stroke, respectively (Federau et al., 2012; Zampini et al., 2020; Zhu et al., 2019).

However, conflicted D* dynamics in ischemic lesions were reported in previous studies. Our results showed that the decreased D* value of infarct lesions was significantly different from that in health contralateral regions (*p* = 0.003), being consistent with the findings reported by Yao et al. (2016). In contrast, comparable D* values were reported in three other studies (Federau et al., 2019; Suo et al., 2016; Zhu et al., 2019), although they all showed a tendency of D* decrease in the affected regions. This discrepancy might be explained by the following aspects: Firstly, inter-subject difference, such as the partial volume effect of cerebrospinal fluid, might limit the robustness of IVIM derived perfusion parameter of D*(Federau et al., 2012; Wolman et al., 2018; Zhu et al., 2019). Secondly, not standardized scan protocol of IVIM sequence, namely, the selection of b values, might also affect the quantification of D* values for ischemic lesions. Similar to ours, Yao et al. (2016) also applied 15 b values, whereas the b values used in other three studies were 5, 6 and 9 respectively (Federau et al., 2019; Suo et al., 2016; Zhu et al., 2019).

To minimize the potential inter-individual impact on the statistical analysis, we further calculated the lesioned/contralateral ratios of each parameter for comparison and correlation analysis between the groups of good outcome and of poor outcome. The present study showed that rADC and rD in the group of poor outcome were significantly lower than those of good outcome by univariate analysis (all *p* < 0.01).

While lesion volume as a confounding factor was controlled, rADC showed a notable negative correlation with day-90 mRS score. This finding was consistent with the results reported in previous acute ischemic stroke studies (Hand et al., 2006; Wardlaw et al., 2002). Furthermore, we also found a strong negative correlation (*r* = −0.731, *p* < 0.001) between rD and day-90 mRS score with lesion volume controlled. As we know, ADC represents the behaviors of water molecule diffusion and microvascular perfusion in tissues (Zhu et al., 2019), while D reveals pure diffusion dynamics of water molecule in tissues (Liu et al., 2019). In addition, our findings revealed a weak negative correlation between rADC and initial neurological function (admission NIHSS score) with lesion volume controlled (*r* = −0.340, *p* = 0.037). Interestingly, this negative correlation was not found between rD and initial neurological function. It is likely that rADC includes more complicated information and better represents the early pathophysiological changes after acute ischemic stroke; however, rD has a stronger negative correlation with clinical outcome.

Previous studies proved that the parameters in perfusion weighted imaging (PWI) such as CBV or time to peak were associated with the clinical outcome after thrombolysis in patients with acute stroke (Nighoghossian et al., 2003; Park et al., 2011). It has also stated that the arterial spin labeling (ASL) derived CBF was related to the clinical outcome of patients with acute stroke treated by intra-arterial thrombectomy (Yoo et al., 2018). In this work, as shown, there was no statistical correlation between cerebral microvascular perfusion related parameter ratios (rD*, rf and rfD*) and initial neurological function, as well as clinical prognosis. The main reasons for this controversy may largely be formulated as follows: First of all, the perfusion parameters of IVIM should be considered as microvascular perfusion, arising mainly from smaller vessels involved in oxygen and metabolites exchange (Yao et al., 2016), while both ASL and conventional PWI are more sensitive to blood flow in larger vessels (Drier et al., 2012). Secondly, microvascular perfusion-related parameters of IVIM are local direct quantitative measurements of ischemic lesions without dependence on precerebral vascular status, such as cervical artery or middle cerebral artery stenosis. In conventional PWI, quantitative computation of blood flow in a voxel requires combining the concentration in the voxel and in the arteries that supply blood to that voxel (Zampini et al., 2020). In terms of ASL technique, there is also a disadvantage of prolonged arterial arrival time which usually leads to an underestimation of CBF (Yoo et al., 2018). Thirdly, the subjects in those previous studies (Arenillas et al., 2002; Nighoghossian et al., 2003; Park et al., 2011; Yoo et al., 2018) underwent thrombolysis or intra-arterial thrombectomy. But, these kinds of patients were excluded in our study. Thus, the subject descrepancy may be responsible for inconsistent results. In general, IVIM-DWI perfusion parameters can be considered a separate effective tool for microperfusion measurement, even though further validation studies are requested in the future.

More recently, two studies raised some issues of current prevalent IVIM model (Wáng, 2021; Xiao & Wáng, 2021). The first issue is that an initial lower S_0_ of the target tissue is associated with a lower D and a higher f. The second issue is that the diffusion component and the perfusion component can’t be completely separated. IVIM fitting model equations results in a certain relationship between D and f. To be specific, if one component, being perfusion component or diffusion component, changes toward one direction (*i.e*., increase or decrease), the other component will be constrained to change toward the opposite direction to a certain extent. These two issues are doomed by the IVIM fitting model equations. We think these two issues may indeed affect the accurate detection of D value and f value by IVIM to a certain extent. However, for studies that do not require precise measurements, but are carried out by studying parametric changing rules, they are less affected. Our study focused on the association of diffusion component and perfusion component with initial neurological function and clinical outcomes in stroke patients, respectively. Therefore, we believe that these two issues will not have much influence on the results of our study. We agree with these two studies (Wáng, 2021; Xiao & Wáng, 2021), and we also think that these two issues are not easily solvable by a better fitting approach, by high signal-to-noise images, or by an extensive array of b-value images. Further research to better separate diffusion component and perfusion component should be pursued.

Several limitations have to be considered in this study. First, this study only focused on acute ischemic stroke but without hyperacute or subacute ischemic stroke. The variations of IVIM parameters in patients with different infarction phases need to be further explored. Second, there are many complicated factors associated with long-term outcome of stroke. The present study made efforts to standardize the baseline clinical characteristics as much as possible by including only first ever patients with supratentorial stroke, executing the most optimal clinical treatment, and eliminating patients with endovascular treatment. Third, the effects of lesion distribution on the neurological function and clinical outcome were not evaluated. In addition, the cohort studied is small, which could be solved in future.

## Conclusion

In conclusion, IVIM derived diffusion dynamics parameters were lower in patients with poor outcome than those in patients with good outcome. Also, significant negative correlations were revealed between IVIM derived diffusion dynamics parameters and initial neurological function as well as clinical outcome in patients with acute stroke. Although, rADC includes more complicated information and better represents the early pathophysiological changes after acute ischemic stroke, rD has a stronger negative correlation with clinical outcome. With these, IVIM can be therefore suggested as an effective non-invasive method for evaluating the initial neurological function and clinical outcome in acute ischemic stroke.

## Supplemental Information

10.7717/peerj.12196/supp-1 Supplemental Information 1 Raw data: Cases 1-10.

10.7717/peerj.12196/supp-2 Supplemental Information 2 Raw data: Cases 11-20.

10.7717/peerj.12196/supp-3 Supplemental Information 3 Raw data: Cases 21-29.

10.7717/peerj.12196/supp-4 Supplemental Information 4 Raw data: Cases 30-39.

10.7717/peerj.12196/supp-5 Supplemental Information 5 Clinical data and IVIM parametric values.

## Additional Information and Declarations

### Data Availability

The following information was supplied regarding data availability: The raw data are available as Supplemental Files.
